# Mitochondrial transport in neurons and evidence for its involvement in acute neurological disorders

**DOI:** 10.3389/fnins.2023.1268883

**Published:** 2023-10-12

**Authors:** Dengfeng Lu, Yun Feng, Guangjie Liu, Yayi Yang, Yubo Ren, Zhouqing Chen, Xiaoou Sun, Yixiang Guan, Zhong Wang

**Affiliations:** ^1^Department of Neurosurgery & Brain and Nerve Research Laboratory, The First Affiliated Hospital of Soochow University, Suzhou, Jiangsu, China; ^2^Suzhou Medical College of Soochow University, Suzhou, Jiangsu, China; ^3^Department of Neurosurgery, Hai’an People’s Hospital Affiliated of Nantong University, Nantong, Jiangsu, China

**Keywords:** kinesin, dynein, myosin, miro, TRAK, acute neurological disorders

## Abstract

Ensuring mitochondrial quality is essential for maintaining neuronal homeostasis, and mitochondrial transport plays a vital role in mitochondrial quality control. In this review, we first provide an overview of neuronal mitochondrial transport, followed by a detailed description of the various motors and adaptors associated with the anterograde and retrograde transport of mitochondria. Subsequently, we review the modest evidence involving mitochondrial transport mechanisms that has surfaced in acute neurological disorders, including traumatic brain injury, spinal cord injury, spontaneous intracerebral hemorrhage, and ischemic stroke. An in-depth study of this area will help deepen our understanding of the mechanisms underlying the development of various acute neurological disorders and ultimately improve therapeutic options.

## 1. Introduction

Mitochondria act as “generators,” producing more than 90% of the energy required for the normal functioning of neurons, and are central to metabolism and bioenergy conversion (Millecamps and Julien, 2013; Lin and Sheng, 2015; Devine and Kittler, 2018). In addition, mitochondria also play an essential role in other cellular processes, such as calcium buffering, neurotransmitter metabolism, action potential formation, synaptic transmission, and short-term plasticity, as well as in promoting cell survival (Tang and Zucker, 1997; Kang et al., 2008; Devine and Kittler, 2018). Therefore, precise regulation of mitochondrial transport and distribution is essential to ensure that mitochondria can be delivered and localized to the areas where they are needed.

The highly polarized morphology is an important feature that distinguishes neurons from other cells. Neurons comprise three parts: the soma, a thin and long axon, and thick and short dendrites with numerous branches (Lin and Sheng, 2015). The mitochondria in these regions are not evenly distributed due to different metabolic demands (Hollenbeck and Saxton, 2005). Synapses, growth cones, axonal branching sites, and Ranvier nodes, which are metabolically active and demanding enormously for ATP, tend to have more mitochondria distributed (Zhang et al., 2010; Course and Wang, 2016). Although mitochondria can be generated locally within axons, it is generally accepted that most are formed within the soma. Damaged mitochondria head back to the cell body to be degraded by the autophagy-lysosome system. Although there is little direct evidence for this hypothesis, it seems uncontroversial considering that the organelles and raw materials required for protein production and degradation are primarily localized in the soma. Thus, the limited extent of mitochondrial biosynthesis and axon degradation challenges neuronal control of distal mitochondrial quality (Sheng and Cai, 2012).

To address this challenge, neurons have evolved finely regulated transport systems based on the cytoskeleton. The cytoskeleton provides the support and backbone for the neuron, maintaining its highly specialized structure and allowing for the efficient transport and stable docking of organelles within the neuron. The neuronal cytoskeleton consists mainly of microtubules and actin filaments (Xiao et al., 2016; Dogterom and Koenderink, 2019). Imaginatively, Microtubules are recognized as “highways with bidirectional lanes” in neurites. Cargoes such as organelles and vesicles can be efficiently transported in both directions by the “truck”-transporter complex, like a car going back and forth on a highway. In the axon, microtubules are evenly arranged with their minus ends oriented toward the soma and plus ends toward the terminus. Newly born mitochondria in the cytosol are delivered to the distal axon via anterograde transport (away from the soma) to provide energy. In contrast, injured mitochondria are repaired through fusion or removed by autophagy via retrograde transport (toward soma). In neurons with high glutamylated microtubules, the average speed and time of a single run did not change in either direction, while the overall motility of mitochondria decreased (Bodakuntla et al., 2020). Unlike the uniform polarity of axonal microtubules, dendritic microtubules exhibit mixed polarity, and therefore, the direction of mitochondrial transport in dendrites may vary depending on microtubule polarity (Zhou et al., 2016; Zheng Y.-R. et al., 2019). Unlike microtubules, actin filaments are more similar to “country roads” just before reaching the terminal. The actin cytoskeleton is abundant in cellular compartments closely related to synapses, such as presynaptic terminals and dendritic spines, creating conditions for the short-distance movement of organelles such as mitochondria and cytoplasmic vesicles at these sites (Langford, 2002).

Mitochondria must be coupled to motor proteins (similarly, loading cargoes onto trucks) to allow polarized transport. Long-distance mitochondrial transport is mainly coordinated by microtubule-based motor proteins, among which the kinesin family mediates anterograde transport directed to the distal end. At the same time, dynein facilitates retrograde transport toward the proximal end (usually the soma). Meanwhile, the actin cytoskeleton and myosin motors direct the movement and anchoring of mitochondria over short distances (Quintero et al., 2009; López-Doménech et al., 2018). The driving force for transporting these motors comes from the hydrolysis of ATP produced by mitochondrial respiration (Hirokawa et al., 2010; Zala et al., 2013). Visual time-lapse imaging methods allow the observation of dynamic, bidirectional transport of neuronal mitochondria along neuronal protrusions, during which they frequently change orientations, pause, or switch to a continuously anchored state. These complex movement patterns are the result of a combination of mitochondria with bidirectional motors and docking and anchoring mechanisms. Mitochondria attach to motors through outer membrane receptors linking to adapter proteins. This receptor-adapter-motor complex enables the precise regulation of targeting and mobility of mitochondrial transport (Kang et al., 2008; Sheng and Cai, 2012).

## 2. Motors

**Figure 1:**
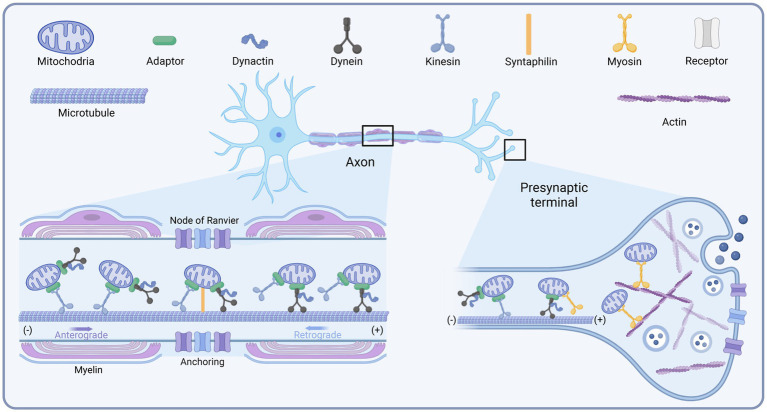
Schematic diagram of the mitochondrial transport motors in neurons. Created with BioRender.com.

It was shown that the transport of mitochondria along microtubules in neurons requires the joint participation of motors and adapters. The different motors are summarized below (Figure 1). Factors reported to influence mitochondrial transport have been summarized in Table 1.

**Table 1:** Factors that have been reported to influence mitochondrial transport.

Factors	Results
Disruption of the KIF5-mitochondrial coupling (Lin and Sheng, 2015; Devine and Kittler, 2018)	Inhibit anterograde transport
Targeted deletion of KIF5A or KIF5B (Kang et al., 2008; Millecamps and Julien, 2013)	
KIF1B-α and KLP6 mutations (Tang and Zucker, 1997)	
Inhibition of the binding of KIF5B to microtubules (Hollenbeck and Saxton, 2005)	
Glucocorticoid receptor translocation to mitochondria (Course and Wang, 2016)	
Disruption of Miro or Milton (Zhang et al., 2010; Sheng and Cai, 2012)	
Silencing syntabulin (Xiao et al., 2016)	
Overexpression of tau (Dogterom and Koenderink, 2019)	
Inhibit HIF-1α inhibitor (Bodakuntla et al., 2020)	
Mutation in TMEM230 (Zheng Y.-R. et al., 2019)	
Destruction of dynactin complexes (Xiao et al., 2016)	Inhibit bidirectional transport
Knock down of Armcx3 (Zhou et al., 2016)	
Administration of the RAR-β agonist CD2019 (100 nM for 72 h) (Bodakuntla et al., 2020)	Facilitate anterograde transport
Knockdown of Myo6 (Langford, 2002)	Facilitate retrograde transport
Consumption of Myo5 (Langford, 2002)	Facilitate mitochondrial motility
Upregulation of Miro1 (Quintero et al., 2009)	
Upregulation of Armcx1 (López-Doménech et al., 2018)	
Knockdown of SNPH (Hirokawa et al., 2010)	
Knockdown of TRAK1 (Pilling et al., 2006; Zala et al., 2013)	Inhibit mitochondrial motility
Knockdown of Armcx1 (López-Doménech et al., 2018)	
Inhibit MTX-2/Miro1/MTX-1/KLC-1 complex (Cai et al., 2005)	
Overexpression of SNPH (Xiao et al., 2016)	
Recruitment of SNPH to mitochondria (Hurd and Saxton, 1996)	
High concentrations of Ca^2+^ (Xia et al., 2003; Karle et al., 2012)	
High glucose concentrations (Nangaku et al., 1994)	

### 2.1. Kinesin

Among the kinesin family members, kinesin-1, also termed KIF5, is the primary driver of the distal distribution of neuronal mitochondria (Pilling et al., 2006). Kinesin contains two heavy chains (KHC) and two light chains (KLC) (Sheng and Cai, 2012). The amino terminus of the heavy chain of kinesin-1 is the motor domain with ATPase and binding directly to microtubules, while its carboxy terminus is the cargo-binding domain, tethering to mitochondria by binding to the adapter proteins Miro and Milton/TRAK (Hirokawa et al., 2010). There are three isoforms of the mammalian KIF5 motor, KIF5A, KIF5B, and KIF5C. KIF5B is widely expressed in various cell types, whereas KIF5A and KIF5C are only distributed in neurons and mediate the transport of membrane organelles such as mitochondria (Hirokawa et al., 2010). Disruption of the KIF5-mitochondrial coupling in hippocampal neurons impaired mitochondrial transport, leading to decreased mitochondrial distribution in distal axons (Cai et al., 2005). This was also verified in Drosophila (Hurd and Saxton, 1996). Targeted deletion of KIF5A or KIF5B also impaired mitochondrial transport, leading to mitochondrial accumulation in the cytosol (Xia et al., 2003; Karle et al., 2012). In addition to KIF5, Kinesin-3 (KIF1B-α) and Kinesin-like protein 6 (KLP6) are also involved in mitochondrial transport (Nangaku et al., 1994). KIF1B-α and KLP6 mutations decreased the average velocity and distal distribution of axonal mitochondria (Tanaka et al., 2011). In muscle cells, Dynamin-related protein 1 (Drp1), A GTPase protein widely distributed in the cytoplasm, binds specifically to KLC1, releasing KIF5B and enhancing microtubule-dependent transport of mitochondria, increasing the speed and distance of mitochondrial transport (Giovarelli et al., 2020). Inhibition of the binding of the molecular motor KIF5B to microtubules and mitochondrial communication along axons inhibits the movement of mitochondria toward the distal axonal segment, resulting in a mitochondrial deficiency in this region (Zorgniotti et al., 2021). Stress, such as sudden trauma, induces alterations in the microtubule network through glucocorticoid signaling pathways. Glucocorticoid receptor translocation to mitochondria induces ER-mitochondrial system retention. Glucocorticoids trigger microtubule dysfunction and kinesin-1 detachment by reducing mitochondrial transport to the pericellular periphery (Choi et al., 2018).

### 2.2. Dynein

Cytoplasmic dynein is the motor driving retrograde mitochondrial transport in axons. While in dendrites, where microtubules are mixed polar, it is involved in mitochondrial transport toward both the distal end and the soma (Sheng and Cai, 2012). Only one dynein has been identified up to now. Dynein contains multiple subunits, including two catalytic dynein heavy chains (DHC), several dynein intermediate chains (DIC), dynein light intermediate chains (DLIC), and dynein light chains (DLC), functioning in coordinating cargo binding or regulating motility. The carboxyl terminus of DHC is the motor domain that enables motility (Lin and Sheng, 2015). Dynactin is a large protein complex with 11 subunits. It binds directly to dynein and microtubule through its p150^Glued^ subunit, thereby enhancing the persistence of dynein motility or regulating its interaction with cargoes (King and Schroer, 2000). Mutations in dynein decreased the distance and duration of retrograde mitochondrial transport in long motor neurons. In contrast, the destruction of dynactin complexes did not undermine the adhesion of motors to the membrane. However, it damaged both anterograde and retrograde transport, suggesting that dynactin is involved in regulating bidirectional transport (Lin and Sheng, 2015). The dynein-dynactin motor complex can move in both directions but toward the minus end of the microtubule in general (Mallik et al., 2005; Ross et al., 2006). This may confer the ability of the dynein to bypass obstacles during intracellular transport.

### 2.3. Myosin

Compared to kinesin and dynein, much less research has been done on myosin. There are 18 classes of myosin (Foth et al., 2006). Myosin drives short-distance transport of organelles and vesicles along actin filaments in presynaptic terminals and growth cones (Sokac and Bement, 2000; Quintero et al., 2009). It was reported that myosin-19 (Myo19) serves as a motor for actin-based mitochondrial motility in vertebrate cells (Quintero et al., 2009). Myo19 is widely expressed in various cell types, including neurons, and its 970 aa heavy chain consists of a motor domain, three IQ motifs, and a short tail. Knockdown analysis suggests that the Myo19 tail is necessary and sufficient for mitochondrial localization. Another study showed that myosin-5 (Myo5) is one of the candidate motors directing mitochondrial motility, consisting of a motor domain, a stem domain, and a tail domain (Sheng and Cai, 2012). Since Myo5 may form a transport complex by interacting with dynein, this probably helps to coordinate long-range transport and short-range movement of mitochondria (Naisbitt et al., 2000; Sheng and Cai, 2012). Similarly, a study in *Drosophila melanogaster* neurons suggested that Myo5 and myosin-6 (Myo6) regulate axonal mitochondrial transport (Pathak et al., 2010). Consumption of Myo5 increased mitochondrial velocity in both directions, while knockdown of Myo6 induced a selective increase in retrograde transport in axons. These findings indicate that Myo5 and Myo6 may compete with microtubule-based motors or that myosin can facilitate mitochondrial docking along actin by moving mitochondria away from microtubule tracks (Sheng and Cai, 2012). However, this needs further confirmation from subsequent studies.

## 3. Adaptors

**Figure 2:**
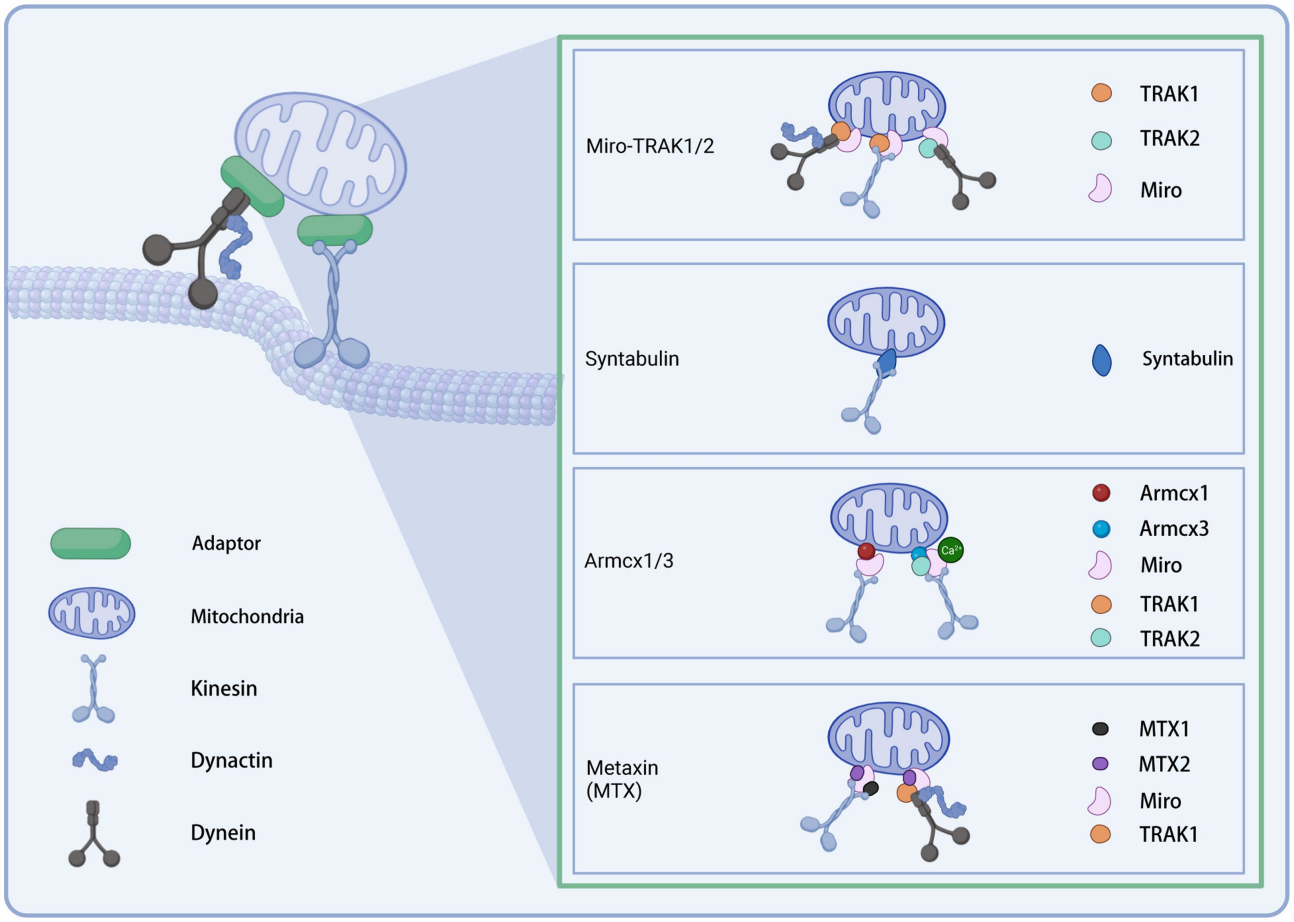
Illustration of the mitochondrial transport adaptors in neurons. Created with BioRender.com.

Distinct motors may require different adaptors to cooperate to function as transporters. The current understanding of adaptors is presented below (Figure 2).

### 3.1. Miro-Milton/TRAK

Mitochondria recruit motor proteins through membrane adapter proteins to allow their motility (Stowers et al., 2002; Kumar et al., 2016). The adapter complex contains two components: Miro and Milton in Drosophila (the homolog in mammals is TRAK) (Cai et al., 2005; Guo et al., 2005; Li et al., 2009). Miro is a member of the Rho GTPase family and contains two EF-arm Ca^2+^ binding motifs and two GTPase domains (Frederick et al., 2004; Fransson et al., 2006; Klosowiak et al., 2013). As an outer mitochondrial membrane receptor, Miro interacts with the motor adapter Milton (or TRAK) to recruit the KIF5 motor to the mitochondrial surface (Lin and Sheng, 2015). GTP hydrolysis or changes in calcium binding by the EF arm may regulate axonal mitochondrial motility in response to calcium signaling and synaptic activity by modulating the Miro-Milton-KIF5 complex (Cai and Sheng, 2009). In mammalian cells, there are two Miro homologous proteins, Miro-1 and Miro-2, with 60% identical sequences, and two TRAKs: TRAK1 and TRAK2. The two TRAK proteins have different functions to direct polarized mitochondrial transport. TRAK1 is mainly localized in axons, while TRAK2 in dendrites. Further, TRAK1 binds to kinesin and dynein and is responsible for axonal mitochondrial transport. In contrast, TRAK2 interacts with dynein and bears responsibility for the dendritic distribution of mitochondria (van Spronsen et al., 2013). In hippocampal neurons, Miro1 is the leading mitochondrial receptor site for TRAK2 (MacAskill et al., 2009). Up-regulation of Miro1 expression enhanced the recruitment of TRAK2 and KIF5 to mitochondria, thereby facilitating mitochondrial motility. In hippocampal neurons, the knockdown of TRAK1 leads to impaired axonal mitochondrial transport, suggesting that the TRAK family plays a vital role in regulating mitochondrial motility (Brickley and Stephenson, 2011). Cells carrying pathogenic variants in TRAK1 exhibit irregular mitochondrial localization and mitochondrial dysfunction, manifesting as altered mitochondrial membrane potential and decreased metabolic state and mitochondrial oxygen consumption (Barel et al., 2017). Disruption of Miro or Milton impaired the distal distribution of axonal mitochondria, resulting in a reduction in the number of mitochondria at distal synapses (Stowers et al., 2002; Guo et al., 2005). There is evidence that Miro can also act as a receptor for dynein (Russo et al., 2009; Nguyen et al., 2014). Milton/Miro complex interacts with dynein to coordinate the relative movement of bidirectional motors (Lin and Sheng, 2015). Interestingly, the absence of dMiro in Drosophila did not wholly block mitochondrial motility: a small number of mitochondria are still located in the neurites (Guo et al., 2005; Russo et al., 2009). An incomplete dependence on Miro1 was found for the distribution of axonal mitochondria (López-Doménech et al., 2016). In Miro1/2 double knockout cells, TRAK1/2 is still recruited to the outer mitochondrial membrane to drive mitochondrial transport (López-Doménech et al., 2018). These data suggest that Miro is not the only adaptor connecting mitochondria to motors. Adaptors other than Miro may exist to recruit motors to transport mitochondria.

### 3.2. Syntabulin

Syntabulin is another KIF5 adapter whose C-terminal transmembrane domain allows mitochondrial targeting. Unlike Miro, which is indirectly attached to the KIF5 motor by association with TRAK, syntabulin directly interacts with the KIF5 cargo binding domain, which mediates the binding of the KIF5 motor to mitochondria (Mutsaers and Carroll, 1998; Su et al., 2004; Cai et al., 2005). In cultured hippocampal neurons, the knockdown of syntabulin or mutation of the KIF5 binding domain to turn off syntabulin-KIF5 coupling resulted in the accumulation of mitochondria in the soma and a reduction in distal mitochondrial distribution (Lin and Sheng, 2015). Analysis of neuronal mobility showed that silencing syntabulin inhibited anterograde mitochondrial transport without affecting retrograde transport (Lin and Sheng, 2015). Together, the above evidence suggests that syntabulin acts as a KIF5 motor adapter to mediate anterograde mitochondrial transport.

### 3.3. Armcx1

Armcx1 (ARMadillo repeat-Containing proteins on the X chromosome 1, also known as Alex1) is a mammalian-specific gene encoding a mitochondria-localized protein. It contains a mitochondrial targeting sequence. Armcx1 is localized on the outer mitochondrial membrane (OMM) of neurons and binds to Miro1 to function. Upregulation of Armcx1 was reported to promote neuronal survival and repair of injured axons after optic nerve injury via augmenting mitochondrial trafficking in mature retinal ganglion cells, dependent on its mitochondrial targeting sequence. In contrast, the knockdown of Armcx1 exacerbated axonal lesions and the death of neurons (Cartoni et al., 2017). The proofs above indicate that Armcx1 regulates mitochondrial transport during neuronal repair.

### 3.4. Armcx3

As another member of the GPRASP (GPCR-associated sorting protein)/ARMCX protein family, the Armcx3 (or Alex3) is highly expressed in the adult nervous system. It has been reported to be involved in mitochondrial dynamics by regulating the speed and distance of mitochondrial movement (López-Doménech et al., 2012). In cultured hippocampal neurons knocked out of endogenous Armcx3, mitochondria moved at reduced velocity and lengths in both anterograde and retrograde directions and are smaller than controls. However, neither the rate nor the distance covered by a single movement of individual mitochondria was affected. Immunofluorescence analysis and immunoprecipitation of transfected HEK293AD cells showed that Armcx3 strongly co-localized with Miro1/2 and TRAK2 rather than KIF5. These data suggest that Armcx3 participates in the KIF5/Miro/TRAK2 transport complex by directly interacting with Miro1-2/TRAK2, consequently regulating mitochondrial dynamics. Interestingly, when Armcx3 was cotransfected with Miro1 mutants lacking the EF-hand structure responsible for Ca^2+^ binding, the Miro1/Armcx3 interaction was greatly diminished, suggesting that this interaction is regulated by Ca^2+^.

### 3.5. Metaxin

Metaxin (MTX) is a class of OMM proteins previously known for its role as a component of a preprotein import complex in the OMM of the mammalian (Armstrong et al., 1997). A recent study in Cryptobacterium hidradenum found that MTX-1/2 facilitated neuronal mitochondrial transport from the soma to dendrites and axons, in which Miro1 and kinesin light chain (KLC-1) played a role. This work indicated that the MTX-2/Miro1/MTX-1/KLC-1 complex enables kinesin-based mitochondrial transport, while the MTX-2/Miro1/TRAK-1 complex allows dynein-based mitochondrial transport (Zhao et al., 2021). The authors concluded that MTX-2 and Miro1 constitute the adaptor core of both motors, while MTX-1 and TRAK-1 assign the KIF5 and dynein motors, respectively, to both complexes. Furthermore, the loss of the above complex leads to impaired mitochondrial transport in human neurons and is therefore required for human neuronal mitochondrial transport.

## 4. Anchoring proteins

In mature neurons, only 20–30% of mitochondria are motile, and approximately 70% are quiescent (Kang et al., 2008; Chen and Sheng, 2013). Specific mechanisms are needed to maintain mitochondrial arrest, and the “anchoring” model is a well-recognized potential mechanism.

### 4.1. Syntaphilin

An earlier study identified syntaphilin (SNPH) as a “static anchor” for axonal mitochondria (Kang et al., 2008). The intermediate domain of SNPH is the axon sorting sequence that mediates its axonal targeting; its C-terminal and N-terminal microtubule-binding domains enable the binding of SNPH to the OMM and microtubules, respectively. Thus, SNPH acts as a “static anchor,” specifically tethering axonal mitochondria to microtubules and stopping the travel (Kang et al., 2008; Chen et al., 2009; Chen and Sheng, 2013), similar to a jack to lift the car. Knockdown of SNPH in mice resulted in a significant increase in the proportion of motile mitochondria in axons and a reduction of the density of mitochondria within axons (Kang et al., 2008). In contrast, overexpression of SNPH remarkably undermined mitochondrial transport in axons (Lin and Sheng, 2015). Another study revealed that the recruitment of SNPH to mitochondria via optogenetic methods blocked rapid mitochondrial transport in both directions (van Bergeijk et al., 2015). Interestingly, SNPH-mediated mitochondrial anchoring also depends on the kinesin and dynein light chain LC8, stabilizing SNPH-microtubule interactions (Chen et al., 2009; Chen and Sheng, 2013). Furthermore, SNPH contains 12% serine residues and several phosphorylation sites, indicating its “anchoring” can be modulated through multiple signaling pathways. Therefore, SNPH is critical in maintaining axonal and synaptic mitochondrial density via an “anchoring” mechanism under varying conditions (Lin and Sheng, 2015).

### 4.2. Mmr1

Mmr1 (mitochondrial Myo2p receptor-related 1) is a member of the DSL1 family of tethering proteins (Swayne et al., 2011). Localized on mitochondria at the bud tip, Mmr1 forms a complex with Myo2p and is thought to be a mitochondrial adapter for Myo2p in yeast involved in mitochondrial distribution (Itoh et al., 2004). Deletion of Mmr1 impairs mitochondrial translocation to the bud tip in yeast, while the overexpression increases mitochondrial anchoring, neither of which damages the mitochondrial movement frequency or velocity (Itoh et al., 2004; Higuchi-Sanabria et al., 2016). However, a recent study suggests that the function of Mmr1 as a tether may not be as persistent as previously thought because its ubiquitinated degradation mediates the dissociation of mitochondria from Myo2 and prevents mitochondrial accumulation at the bud tip or bud neck (Obara et al., 2022). Altogether, the spatiotemporally regulation of Mmr1 degradation is critical for maintaining the proper distribution of mitochondria in yeast daughter cells. However, the homologous protein of Mmr1p in eukaryotes has not been reported yet.

## 5. Other mechanisms

### 5.1. TMEM230

TMEM230 (transmembrane protein 230) is a recently identified gene associated with PD. Overexpression of WT and mutant TMEM230 or knockdown of the endogenous protein in cultured SH-5Y5Y cells and mouse primary hippocampal neurons impaired retrograde axonal mitochondrial transport and induced neurodegeneration. And the mutant-induced impairment of mitochondrial transport was much more severe. Therefore, the authors concluded that maintaining proper TMEM230 levels could be critical for axonal mitochondrial transport and neuronal survival. These findings provide new insights into the role of TMEM230 in the pathogenesis of Parkinson’s disease (Wang X. et al., 2021). Unlike the chronic course of Parkinson’s disease, acute neurological disorders may be more intense in terms of neuronal stress, and the role of TMEM230 in this remains to be further investigated in depth.

### 5.2. Ca^2+^

Axons can be up to one meter long. Hence, the distribution of mitochondria in neurons as energy-supplying organelles and calcium reservoirs is critical for maintaining axonal morphological and functional homeostasis. Neurons are subjected to repetitive action potentials, which lead to a large influx of Ca^2+^. The distribution of mitochondria along axons can transport ATP and Ca^2+^ to the appropriate places. This activity is mediated by the Miro-Milton complex. High concentrations of Ca^2+^ act as a “stop” signal, causing detachment of the KIF5 motor from microtubules or the Miro/Trak complex, resulting in the arrest of mitochondrial transport (Wang and Schwarz, 2009). Moreover, the increase of Ca^2+^ reduces the ligation frequency and the run length of Myo19, thus inhibiting the movement of mitochondria at synaptic terminals (Pollard et al., 2023). This allows the mitochondria to remain in the metabolically active zone, producing ATP and buffering Ca^2+^. However, a high concentration of Ca^2+^ also increases Drp1 activity, triggering mitochondrial fragmentation and metabolic disorders, thus contributing to axon collapse (Bao et al., 2018). In general, the current evidence seems to suggest that moderately high concentrations of Ca^2+^ play a negative regulatory role in mitochondrial transport.

### 5.3. Glucose

Due to the potentially long span, the glucose concentration may vary at the locations where neurons pass (Matsuda et al., 2009). Mitochondria stay where nutrients such as glucose are most concentrated, as reported in one study – in rat axons, mitochondrial transport was halted at high glucose concentrations (Agrawal et al., 2018). This arrest is mediated by high glucose concentrations via glycosylation of the motor adaptor Milton (TRAK) by the glucose-activated enzyme O-GlcNAc transferase (OGT) (Pekkurnaz et al., 2014). Further studies showed that four and a half LIM domains protein 2 (FHL2) binds to O-GlcNAcylated TRAK, anchoring mitochondria to F-actin and halting its motility (Basu et al., 2021). Disruption of F-actin restores mitochondrial movement. Thus, mitochondrial dynamics can be adapted to changes in glucose concentration within the neuron to improve energy production (Agrawal et al., 2018; Basu et al., 2021).

### 5.4. Tau

Tau is a neuronal microtubule-associated protein (MAP) that promotes the assembly and binding of microtubules and inhibits microtubule dynamics. An injury-dependent increase in neuronal tau acetylation (ac-tau), mediated by S-nitrosylated GAPDH, has been observed in several forms and stages of TBI (Shin et al., 2021). An earlier study demonstrated that tau overexpression resulted in profound alterations in the mitochondrial distribution in differentiated neuroblastoma cells. This was manifested by severe disruption of microtubule-based anterograde transport, while retrograde transport was less affected. Consequently, negative transport predominates and causes mitochondria to aggregate toward the center of the cell (Ebneth et al., 1998).

### 5.5. RAR-β

Retinoic acid receptors (RARs)-β are members of the nuclear receptor superfamily, and evidence for their involvement in neuronal mitochondrial transport is beginning to emerge. Previous studies have shown that increased RAR-β content coincided with axon growth rate in cultured cortical neurons (Corcoran and Maden, 1999; Hoecker et al., 2013). This was confirmed by the elongation of neuronal axons following administration of the RAR-β agonist CD2019 (100 nM for 72 h) (Trigo et al., 2019). Further, tracer imaging revealed that RAR-β recruits mitochondria at the distal end of axons. Once the combination of CD2019 and CAY10585, hypoxia-inducible factor-1 alpha subunit (HIF-1α) inhibitor, was applied, the effect of RAR-β on mitochondrial anterograde transport and axon growth was inhibited. Thus, the authors suggest that RAR-β activation promotes the velocity and amount of anterograde transport of neuronal mitochondria through HIF-1α signaling, promotes mitochondrial proliferation, and induces neurite growth (Trigo et al., 2019). The mitochondrial chaperone GRP75 is known for its involvement in mitochondrial-endoplasmic reticulum coupling. This study also found that RAR-β-mediated mitochondrial recruitment was accompanied by an upregulation of GRP75 and increased co-localization with mitochondria. The interaction between them is thought to be required for neurite elongation. Recently, another report indicated that HIF-1α plays a neuroprotective role by targeting the miR-20a-5p/KIF5A axis to regulate autophagic flux and rescue oxygen–glucose deprivation and reoxygenation (OGD/R)-induced neuronal damage (Cao et al., 2022). The above evidence suggests a non-negligible involvement of RAR-β signaling in mitochondrial transport. However, the exact mechanism remains to be further elucidated.

## 6. Intercellular mitochondrial transfer

The intercellular mitochondrial transfer has received increasing attention in recent years. Intercellular mitochondrial transfer is essential for intercellular communication and maintenance of cell viability. Damaged mitochondria can be transferred from neurons to astrocytes for recycling and disposal (Davis et al., 2014; Hayakawa et al., 2016). Healthy mitochondria can be transferred from astrocytes to injured neurons to help restore homeostasis (Liu et al., 2022). Various pathways, such as tunneling nanotubes (TNTs), extracellular vesicles, and gap junctions, enrich the trans-cellular transfer of mitochondria (Nasoni et al., 2021; Jain et al., 2023). There are many excellent reviews of the intercellular transfer of mitochondria (Shanmughapriya et al., 2020; Fairley et al., 2022; Luchetti et al., 2022), so we will not discuss them here.

## 7. Local disposition of mitochondria

Parkin and PTEN-induced kinase 1 (PINK1) have received much attention in mitophagy, which functions as a critical pathway for mitochondrial quality control (Eldeeb et al., 2022). Given the short half-life (in minutes), synthesizing in the cytoplasm and transporting PINK1 to the distal end (which may take considerable time in neurons with long axons, such as the sciatic nerve) to maintain mitochondrial quality seems to become less practical. Then how does the PINK1-Parkin pathway act distally? Recent studies have shown that PINK1 mRNA is present in axons (Ashrafi et al., 2014). PINK1 mRNA is first co-transported with neuronal mitochondria to axons and then translated. While translating, the mitochondrial outer membrane proteins synaptojanin 2 binding protein (SYNJ2BP) and synaptojanin 2 (SYNJ2) are on the mitochondrial need to bind to PINK1 mRNA through the RNA binding domain of SYNJ2, ultimately triggering mitochondrial autophagy. The above evidence suggested that axonal translation makes distal mitophagy feasible without having to be transported back to the soma (Soumbasis and Eldeeb, 2022). Indeed, selective removal of harmful components from mitochondria has been reported, such as resident proteases in mitochondria (Misgeld and Schwarz, 2017). Defective proteins can also fuse with lysosomes through small vesicles sprouting from mitochondria, known as mitochondria-derived vesicles (MDVs) (Shin et al., 2020). The above mechanisms improve the precision, efficiency, and flexibility of mitochondrial quality control in neurons.

## 8. Mitochondrial fusion-fission dynamics

Healthy mitochondria are tube-shaped, while damaged mitochondria appear spherical. The morphology of mitochondria reflects whether the organelle is healthy and is the result of equilibrium between fission and fusion (Ul Fatima and Ananthanarayanan, 2023). Mitochondrial fission is mediated by the cytosolic GTPase DRP1, whereas fusion by the dynamin-like GTPases Mitofusin 1/2 (Mfn1/2) and the optic atrophy protein 1 (OPA1) (Yue et al., 2014; Herkenne et al., 2020; Zaninello et al., 2020; Sidarala et al., 2022; Rios et al., 2023). Fission allows defective mitochondrial components to be isolated and cleared by mitochondrial autophagy, maintaining the polarized state of mitochondria (Youle and van der Bliek, 2012). DRP1 mutations result in the absence of distal mitochondria, suggesting an essential role in mitochondrial localization (Ishihara et al., 2009). Fusion is initiated by Mfn1/2-regulated fusion of the OMM, followed by OPA1-regulated fusion of the inner mitochondrial membrane (IMM) (Filadi et al., 2018). Fusion allows the exchange of mitochondrial proteins and mitochondrial DNA (mtDNA), which reduces metabolic stress and is one of the pathways to repair damaged mitochondria (Youle and van der Bliek, 2012; Shin et al., 2020). Mitochondrial fission and fusion have been covered in detail in several fascinating reviews and are, therefore, beyond the scope of this review (Burté et al., 2015; Dorn, 2019; Sprenger and Langer, 2019; Valdinocci et al., 2019; Fernandes et al., 2020; Shin et al., 2020; Adebayo et al., 2021; Yang et al., 2021; López-Doménech and Kittler, 2023). Mitochondrial motility has been reported to be a determinant of fusion (Twig et al., 2010). It is noteworthy that Miro1/2, which plays a crucial role in mitochondrial transport, also functions in fusion cessation, demonstrating the multiple actions of Miro1/2 in mitochondrial dynamics (Ul Fatima and Ananthanarayanan, 2023). Dynamic regulation of mitochondrial transport allows neurons to respond rapidly to changes in synaptic activity, and modulation of dynamics makes it possible to adjust metabolic efficiency to accommodate energy demands (López-Doménech and Kittler, 2023). Although how fission/fusion and transport coordinate remains poorly defined, what is certain is that these two processes work together to maintain neuronal energetic homeostasis.

## 9. Evidence for the involvement of mitochondrial transport in acute neurological disorders

Acute neurological diseases include traumatic brain injury, spinal cord injury, and intracerebral hemorrhage (Zhang et al., 2021; Piffer et al., 2022). Stress such as sudden trauma causes tissue destruction in a short time, followed by progressive excitotoxicity, oxidative stress, mitochondrial dysfunction, and energy collapse, eventually leading to cell death (Cheng et al., 2022). Subsequently, a substantial energy supply is required for neural repair after injury. Exploring mitochondrial transport in this context is of great significance, as targeting this process may exert neuroprotective effects by improving mitochondrial dysfunction and alleviating the imbalance between energy supply and demand. Although limited, evidence for the involvement of mitochondrial transport in acute neurological disorders has emerged. These disorders include traumatic brain injury, spinal cord injury, spontaneous intracerebral hemorrhage, and ischemic stroke (see Supplementary Table 1 for details).

### 9.1. Traumatic brain injury

Traumatic brain injury (TBI) is a common acute disease worldwide, often caused by car crashes, athletic accidents, and violent incidents, and it burdens patients, families, and society. TBI consists of primary and secondary injuries (Werner and Engelhard, 2007). Primary injuries occur in the immediate aftermath of an external force and include brain parenchymal deformation, diffuse axonal injury, and intracerebral hematoma (LaPlaca et al., 2007). Secondary injury progressed relatively slowly and involves excitotoxicity, mitochondrial dysfunction, calcium overload, oxidative stress, neuroinflammation, secondary axonal injury, and apoptosis (Wang et al., 2016). The effects of calcium overload on mitochondrial transport have been described previously. The role of microtubule disruption on mitochondrial translocation will be discussed in the next section rather than here. Mitochondrial dynamics, including the extent of fusion, fission, and translocation, is altered following TBI, which has been well summarized by Shin et al. (2020). A recent study reported the role of Miro1 in TBI rats (Chen et al., 2021). An increase in Miro1 expression was first found after TBI. Knockdown of Miro1 inhibited mitochondrial transport, exacerbated neuronal apoptosis and energy deficit, and further aggravated brain edema and neurological dysfunction in rats. These findings suggested that Miro1 might provide neuroprotective effects through augmented mitochondrial transport. Our team explored the role of mitochondrial transport-associated Armcx1 in TBI (Lu D. et al., 2023). It was found that Armcx1 expression was decreased in cortical tissues of TBI mice, and overexpression of it improved neuronal mitochondrial status, attenuated apoptosis, and was associated with better behavioral performance. Again, the critical role of axonal mitochondrial transport in secondary injury after TBI is emphasized. Dementia pugilistica (DP) is a neuropathological alteration following chronic TBI that manifests as a decline in cognitive function. An earlier study suggested that kinesin and dynein levels were significantly decreased in DP patients. The limited evidence suggests a potential association of axonal mitochondrial transport impairment with TBI. More in-depth studies are urgently needed to help us further assess such an important topic.

### 9.2. Traumatic spinal cord injury

Traumatic spinal cord injury (SCI) occurs when an external force damages the spinal cord and leads to neurological dysfunction and disability, with traffic accidents, accidental falls, and violent events as the common causes (Wagner et al., 2018; Freund et al., 2019). Primary injury results from the initial mechanical force on the spinal cord and leads to glutamate excitotoxicity, oxidative stress, breakdown of the brain-spinal cord barrier (BSCB), demyelination, ischemia, and edema (Hellenbrand et al., 2021; Ribeiro et al., 2023). This process initiates a secondary injury cascade that further causes cell death and spinal cord injury. The spinal cord primarily comprises upstream and downstream conduction bundles, that is, long axonal bundles of numerous sensory/motor neurons (Han et al., 2020). The critical role of axonal transport disorders in spinal cord injury is, therefore, self-explanatory. In a mouse model of spinal cord injury, microtubule activity at the proximal end of the dissection is dramatically increased. It develops into retractile bulbs within days, with disorganized polarity of the microtubules inside (Ertürk et al., 2007; Kleele et al., 2014). In this context, the transport of organelles such as mitochondria is disturbed and accumulates at the cut-off ends. Using paclitaxel, a microtubule stabilizer, attenuates axonal damage and axon bulb formation in spinal cord transected mice and promotes functional recovery (Ertürk et al., 2007; Hellal et al., 2011; Kleele et al., 2014). Enhanced mitochondrial transport to support energy demand promotes recovery of injured axons, as demonstrated in SNPH knockout mice (Zhou et al., 2016). Another article reported the role of SNPH in this field (Han et al., 2020). The authors discovered that enhancing axonal mitochondrial transport by deleting SNPH restored injury-induced mitochondrial depolarization. Enhanced regeneration of corticospinal tracts (CST) passing through a spinal cord lesion, accelerated regrowth of monoaminergic axons across a transection gap, and increased compensatory sprouting of uninjured CST in SNPH^−/−^ mice were found in three mouse models, respectively. Axonal regeneration involves reconstruction of the cytoskeleton, synthesis, transport of raw materials, and adequate energy supply to form functional growth cones (Lu Q. et al., 2023). It is reasonable to believe that an in-depth study on mitochondrial transport will be an essential foundation for future SCI repair.

### 9.3. Spontaneous intracerebral hemorrhage

Spontaneous intracerebral hemorrhage (ICH), a rupture of a blood vessel in the brain parenchyma, is the leading cause of death and disability in adults worldwide (Ikram et al., 2012; Kirshner and Schrag, 2021). Secondary injuries induced by ICH lead to pathological changes such as neuronal death and axonal damage (Wagner, 2007; Sangha and Gonzales, 2011). Recently, an article was published reporting the effect of Miro1 on secondary injury after ICH (Li et al., 2021). Overexpression of Miro1 ameliorated MMP depolarization and reduced neuronal damage by promoting mitochondrial transport and distribution, which was validated in cultured oxyhemoglobin (OxyHb)-treated neurons. MEC17 is a specific α-microtubulin acetyltransferase that catalyzes α-microtubulin acetylation (Li and Yang, 2015). Another research demonstrated that mitochondria in neuronal axons and dendrites preferentially bind to acetylated α-microtubulin. α-microtubulin acetylation induced by MEC17 attenuated axonal injury in ICH mice via restoring mitochondrial transport, protecting the integrity of CSTs, and thus promoting fine motor redevelopment (Yang et al., 2022). Netrin −1 is thought to be a diffusible chemokine that attracts or repels axons (Salinas, 2003). In 2018, one team from China reported that modulation of KIF1A-Netrin-1 potentially exerts neuroprotective effects on secondary brain injury after ICH (Wang et al., 2017). Another study by Xu et al. suggested that SNPH knockdown combined with Armcx1 overexpression protected perihematoma brain cells from death and improved neurobehavioral deficits in mice. Considering the complex mechanisms of secondary injury, the above studies targeting mitochondrial transport after ICH provide minimal evidence, and more comprehensive and in-depth studies are urgently needed to clarify the underlying mechanisms.

### 9.4. Ischemic stroke

Ischemic stroke refers to the narrowing or occlusion of cerebral arteries caused by cerebral thrombosis or dislodgement of emboli from other parts of the circulation, resulting in reduced or even blocked cerebral blood flow, which in turn causes ischemia and hypoxia or even necrosis of brain tissue. Ischemic stroke accounts for the primary type of stroke and can cause functional deficits in the corresponding areas, impaired consciousness, and even death (Herpich and Rincon, 2020; Mendelson and Prabhakaran, 2021). Current therapies are mainly based on recanalizing occluded blood vessels, which is insufficient or unavailable for many patients (Wang L. et al., 2021). In oxygen–glucose-deprived neurons, axonal damage is attenuated by enhanced retrograde transport, which may be attributed to mitochondrial autophagy removing harmful mitochondria (Zheng Y. et al., 2019). The role of HIF-1α in ischemic injury was reported recently (Cao et al., 2022). OGD/R increased HIF-1α expression, negatively regulating miR-20a-5p expression by targeting its promoter. Meanwhile, miR-20a-5p directly targets the untranslated region of KIF5A mRNA and inhibits its translation. Ultimately, HIF-1α promotes the expression of KIF5A. The critical role of KIF5A in the anterograde transport of axonal mitochondria has been described previously. It can be inferred that the upregulation of transport motor expression after ischemic injury is a remedial measure by neurons to rescue energy depletion. However, there is considerable doubt as to how much this upregulation can help. Given the lack of evidence, a comprehensive evaluation of this topic is not yet possible.

## 10. Summary and prospects

Mitochondrial transport is essential for maintaining neuronal homeostasis due to the specificity of neuronal polarization morphology and its high demand for energy consumption. In the present context, this importance is manifested in at least two aspects: supplying energy for axonal repair and removing damaged mitochondria through somatic autophagy. However, our understanding of this area is still limited, and many unanswered questions still haunt us. For example, how mitochondria coordinate bidirectional transport of motor proteins, how mitochondrial axonal transport can be precisely coordinated between microtubules and actin microfilaments, and how to coordinate mitochondrial transport with other mitochondrial quality control mechanisms such as mitochondrial autophagy and fusion when mitochondria are damaged. Moreover, in addition to mitochondrial transport, the reconstruction of axons requires various cytoskeletal building blocks, and the generation/transport of these accessories is also an issue that must be considered.

Nevertheless, this field is currently gaining more and more scholarly attention. Acute neurological diseases often initiate rapidly and represent a sudden energy failure. Therefore, attempts at energy salvage, such as mitochondrial transport, are particularly critical. Unfortunately, the current understanding of the mechanism is far from satisfactory. As related research progresses, our knowledge of mitochondrial transport will gradually improve soon, and the development of therapies targeting mitochondrial transport will be on the agenda.

## Author contributions

DL: Conceptualization, Investigation, Methodology, Writing – original draft. YF: Writing – original draft. GL: Data curation, Formal analysis, Investigation, Writing – review & editing. YY: Writing – review & editing, Visualization. YR: Writing – review & editing, Supervision. ZC: Supervision, Writing – review & editing, Resources. XS: Resources, Supervision, Writing – review & editing. YG: Supervision, Writing – review & editing, Validation. ZW: Supervision, Writing – review & editing, Funding acquisition, Resources.
