# Supplementary material for: Mitochondrial transport in neurons and evidence for its involvement in acute neurological disorders
Source: Front Neurosci. 2023 Oct 12;17:1268883. doi: 10.3389/fnins.2023.1268883 (PMC10600463; doi:10.3389/fnins.2023.1268883)
Supplement: Supplementary file 1 [file Table_1.docx]

**Table 1**. Mechanisms of mitochondrial transport reported in acute neurological diseases.

| **Author** | **Time** | **Disease** | **Animal/cell** | **Model** | **Main results** |
| --- | --- | --- | --- | --- | --- |
| Lu et al.^1^ | 2023 | TBI | Mouse, primary mouse cortical neurons | Controlled cortical impact | Armcx1 expression was decreased in cortical tissues of TBI mice, and overexpression of it improved neuronal mitochondrial status, attenuated apoptosis, and was associated with better behavioral performance. |
| Chen et al.^2^ | 2021 | TBI | Rat | Weight-drop model | Miro1 expression was increased after TBI; knockdown of Miro1 inhibited mitochondrial transport, exacerbated neuronal apoptosis and energy deficit, and further aggravated brain edema and neurological dysfunction. |
| Han et al. ^3^ | 2020 | SCI | Mouse | Cervical 5 dorsal hemisection model; unilateral pyramidotomy model; thoracic 8 spinal complete transection model. | Strengthening mitochondrial transport in axons by removing SNPH recovered injury-induced mitochondrial depolarization; SNPH^−/−^ mice displayed enhanced CST regeneration, accelerated regrowth of monoaminergic axons, and increased compensatory sprouting of uninjured CST. Remarkably, regenerated CST axons formed functional synapses that facilitated the recovery of motor function. |
| Li et al.^4^ | 2021 | ICH | Rat; primary rat cortical neurons | ICH model via injecting  self-body heart blood (100 µL) into the right basal ganglia; neurons were treated with OxyHb. | Overexpression of Miro1 decreased necrosis and apoptosis in OxyHb-treated neurons, alleviated MMP depolarization, improved mitochondrial quality, and reduced neuronal injury by promoting mitochondrial transport and distribution, and thus improved brain edema, neurobehavioral deficits and neuronal death in ICH rats. |
| Yang et al. ^5^ | 2022 | ICH | Mouse | ICH model via injecting autologous blood (25 µL) into the striatum. | Depletion of acetylated alpha-tubulin in MEC17 mice exacerbated axonal injury, dysfunction of mitochondrial transport in axons, and motor deficits. In contrast, Increased microtubule acetylation dramatically attenuated axonal injury, maintained the integrity of CST and recovered fine motor function after ICH. |
| Wang et al.^6^ | 2017 | ICH | Rat; primary rat cortical neurons | ICH model induced by autologous  whole blood (100 µL) injection into the basal ganglia; neurons were treated with OxyHb (10µM) in vitro. | Implementation of recombinant netrin-1 mitigated neuronal apoptosis and degeneration in ICH rats. Additionally, KIF1A overexpression increased concentrations of netrin-1 in CSF and cell culture supernatant and acted as a neuroprotective agent through netrin-1 and its receptor pathways. |
| Xu et al.^7^ | 2023 | ICH | Mouse; primary mouse cortical neurons | ICH model was created through the injection of 30 μl of autologous blood into the basal ganglia; neurons were exposed to OxyHb(10 μM) | SNPH knockdown combined with Armcx1 overexpression protected perihematoma brain cells from death and improved neurobehavioral deficits in mice. |

Note: TBI, Traumatic brain injury; Miro1, mitochondrial Rho GTPase 1; SCI, spinal cord injury; SNPH, syntaphilin; CST, corticospinal tract; ICH, intracerebral hemorrhage; OxyHb, oxygen hemoglobin; MMP, mitochondrial membrane potential; CSF, cerebrospinal fluid.

**References**

1. Lu, D.; Wang, Y.; Liu, G.; Wang, S.; Duan, A.; Wang, Z.; Wang, J.; Sun, X.; Wu, Y.; Wang, Z., Armcx1 attenuates secondary brain injury in an experimental traumatic brain injury model in male mice by alleviating mitochondrial dysfunction and neuronal cell death. *Neurobiol Dis* **2023,** *184*, 106228.

2. Chen, C.; Lu, L.; Zhu, J.; Gu, X.; Liu, B.; Li, D.; Chen, G., Miro1 provides neuroprotection via the mitochondrial trafficking pathway in a rat model of traumatic brain injury. *Brain Research* **2021,** *1773*, 147685.

3. Han, Q.; Xie, Y.; Ordaz, J. D.; Huh, A. J.; Huang, N.; Wu, W.; Liu, N.; Chamberlain, K. A.; Sheng, Z.-H.; Xu, X.-M., Restoring Cellular Energetics Promotes Axonal Regeneration and Functional Recovery after Spinal Cord Injury. *Cell Metab* **2020,** *31* (3).

4. Li, B.; Zhang, Y.; Li, H.; Shen, H.; Wang, Y.; Li, X.; Cui, G.; Chen, G., Miro1 Regulates Neuronal Mitochondrial Transport and Distribution to Alleviate Neuronal Damage in Secondary Brain Injury After Intracerebral Hemorrhage in Rats. *Cellular and Molecular Neurobiology* **2021,** *41* (4), 795-812.

5. Yang, Y.; Chen, X.; Feng, Z.; Cai, X.; Zhu, X.; Cao, M.; Yang, L.; Chen, Y.; Wang, Y.; Feng, H., MEC17-induced α-tubulin acetylation restores mitochondrial transport function and alleviates axonal injury after intracerebral hemorrhage in mice. *J Neurochem* **2022,** *160* (1), 51-63.

6. Wang, J.; Zhai, W.; Yu, Z.; Sun, L.; Li, H.; Shen, H.; Li, X.; Liu, C.; Chen, G., Neuroprotection Exerted by Netrin-1 and Kinesin Motor KIF1A in Secondary Brain Injury following Experimental Intracerebral Hemorrhage in Rats. *Frontiers In Cellular Neuroscience* **2017,** *11*, 432.

7. Xu, X.; Li, H.; Lu, S.; Shen, Y., Roles of syntaphilin and armadillo repeat-containing X-linked protein 1 in brain injury after experimental intracerebral hemorrhage. *Neuroscience Letters* **2023,** *809*, 137300.
